# Editorial: The Gut Microbiota Orchestrates the Neuronal-Immune System

**DOI:** 10.3389/fcell.2021.672685

**Published:** 2021-03-29

**Authors:** Paola Brun, Hamid I. Akbarali, Ignazio Castagliuolo

**Affiliations:** ^1^Department of Molecular Medicine, University of Padova, Padova, Italy; ^2^Department of Pharmacology and Toxicology, Virginia Commonwealth University, Richmond, VA, United States

**Keywords:** gut microbiota, inflammation, gut dysmotility, dysbiosis, neuroimmune cross-talk

The Research Topic “The Gut Microbiota Orchestrates The Neuronal-Immune System” was proposed to bring together studies addressing the role of intestinal microbes in determining the homeostasis or the disease both at local and systemic levels. The gut microbiota is the richest and most complex microbial ecosystem in the human body as it is made up of trillions of cells, including bacteria and fungi, whose collective genetic material significantly impacts on host's functions. Moreover, in the gut, unlike in other body compartments, the microbial community is in close contact with enterocytes, enteric neurons, and immune cells. Consolidate knowledge has clarified that alterations in gut microbiota, namely dysbiosis, influence intestinal motility, mucosal permeability, and antigen tolerance. The direct interaction between neuronal and immune cells has been extensively investigated in the last few years, establishing the “neuro-immune cell unit” as the local checkpoint in controlling intestinal functions. However, studies merging the multifaceted role of the gut microbiota in the neuro-immune units are still limited.

In the gut wall, immune cells face food antigens, symbiotic and potentially pathogenic microbes and set a tolerance threshold to prevent aberrant inflammation. Loss of tolerance results in abnormal activation of the immune response and is involved in local disorders ranging from inflammatory bowel diseases to irritable bowel syndrome. In this scenario, the central nervous system interacts with the intestinal immune system to modulate inflammation through humoral and neural pathways, using a mechanism referred to as the intestinal cholinergic anti-inflammatory pathway. On the other side, gut microbes drive the secretion of neuroactive factors to set the enteric neuronal network's activation and function. Thus, the gut microbiota is on the fringes of the neuronal-immune system and gut dysbiosis has dramatic consequences for tissue homeostasis, resulting in intestinal inflammatory diseases and dysmotility.

We are grateful to the 54 Authors from three continents that contributed to this Research Topic with review and original research. Their publications consider the key events orchestrating the impact of the gut microbiota in intestinal and systemic disorders, looking at the fundamental mechanisms and pathways behind the mere alterations in microbiota composition. The main results are outlined in Figure 1. The contributions to this Research Topic also clarify the impact of host genetic factors and soluble neurotransmitters in the microbiota-gut-brain axis, paving the way for potential modulators in inflammatory conditions and disorders of the central nervous system. This collection of publications widens knowledge in the field and provides novel insights in supporting experimental models worth considering in future research. In the following, we give an overview of the research articles included in the Research Topic.

**Figure 1:**
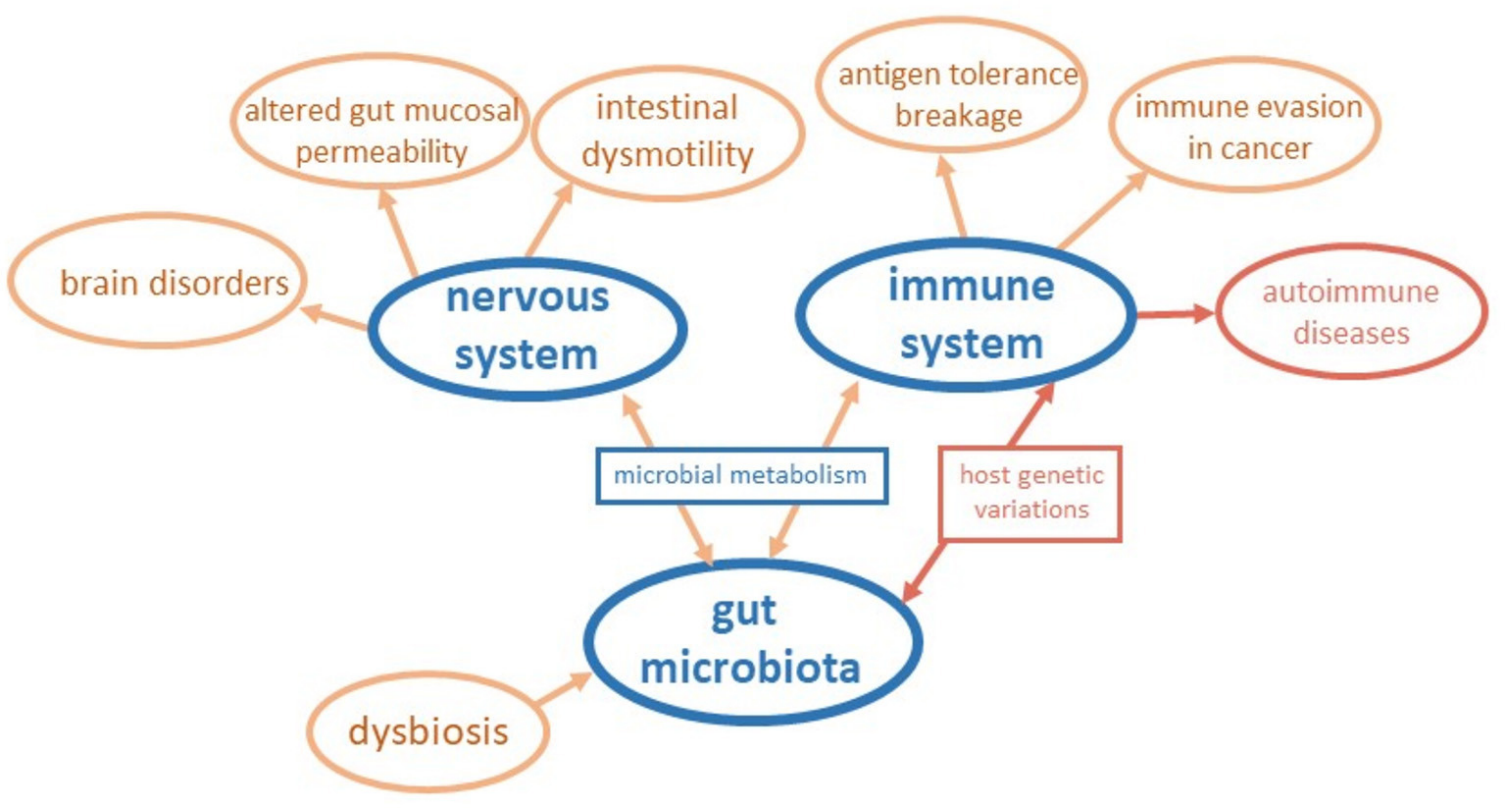
Schematic representation of insights coming from contributions to the Research Topic. The connections among the gut microbiota, the nervous system, and the immune system are outlined considering the main results of papers of the Research Topic.

Dehhaghi et al. reviewed the gut-brain bidirectional pathway in neuroinflammation and brain tumors. They provided state-of-the-art information supporting the involvement of gut microbiota in brain cancers via (i) the kynurenine metabolism, (ii) deprivation of amino acids due to the metabolism of the gut microbiota, and (iii) the microglia dysfunction mediated by immune cells. In the gut, bacteria can reduce the systemic availability of tryptophan and arginine, utilizing them to produce microbial proteins or directing them into microbial metabolic pathways. In humans, the kynurenine pathway is the main route of tryptophan metabolism, and dysregulation of the kynurenine pathway has been linked with cancer development because of the disruption of the antitumoral immune responses. By drawing the complexity of the gut microbiota-brain cancer axis, this review deepens our understanding of microbial metabolism's role in shaping the anti-tumor immune responses, inspiring the development of novel therapeutic strategies.

Metabolites generated in the gut by dysbiotic microbiota modulate brain function with different outcomes and have been correlated with brain disorders. Patients who have schizophrenia have dysregulated microbial profiles. However, clinical data lack consistency and animal models of schizophrenia did not recapitulate gastrointestinal dysfunction or dysbiosis, making the research difficult. Gubert et al. performed gut microbiome profiling in metabotropic glutamate receptor 5 knockout (mGlu5KO) mice, a preclinical model of schizophrenia. By 16S rRNA sequencing of bacterial genomic DNA from fecal samples, they found a significant genotype difference in microbial beta diversity with decreased relative abundance in the *Erysipelotrichaceae* family and *Allobaculum* genus. The differential community composition substantiates the utility of the mGlu5KO animal model for the investigations of gut dysbiosis and related gut microbiota-brain axis signaling as a potential modulator in schizophrenia. Indeed, bacteria in the gut secrete soluble factors that positively influence the psychological status of the host. In their research paper, Beck et al. identified one *Lactobacillus* strain isolated from newborns as psychobiotic. Indeed, the strain has an anti-inflammatory effect (increases production of IL-10), modulates gut microbiota composition (enhances the relative presence of *Bacteroidetes*), and contributes to the gut-brain axis by augmenting serum dopamine level in healthy mice.

Increasing evidence suggests a role for the gut microbiome in autoimmune diseases, including multiple sclerosis, but the impact of host genetic variation in shaping the gut microbiota has been largely overlooked. Kishikawa et al. performed a phylogenetic, functional gene, and pathway analysis of the gut microbiome of Japanese patients with relapsing-remitting multiple sclerosis revealing novel interactions among the gut metagenome and the host genome. In particular, they identified discrepancies in the case-control comparison for gene clades related to immune system function and molecular pathways involved in recognizing lipopolysaccharide. As a result, patients with multiple sclerosis have prompt secretion of inflammatory cytokines during disease relapses, thus highlighting the role of the gut microbiome in the onset and long-term evolution of multiple sclerosis. Alteration in immune response and aberrant recognition of microbial antigens drive functional gastrointestinal disorders such as irritable bowel syndrome (IBS). Arredondo-Hernandez et al. analyzed host single nucleotides polymorphisms (SNPs) and taxonomic composition of the gut microbiota collected from IBS patients and healthy controls selected among Mestizo Latin America, a population suffering from several inflammatory disorders. They identified 76 SNPs and a relative decrease in *Bacteroidetes* in IBS patients that may serve as a hallmark in IBS diagnosis.

## Author Contributions

All authors listed have made a substantial, direct and intellectual contribution to the work, and approved it for publication.

## Conflict of Interest

The authors declare that the research was conducted in the absence of any commercial or financial relationships that could be construed as a potential conflict of interest.

